# Investigating SARS-CoV-2 Neutralising Antibody Response in Sheep

**DOI:** 10.3390/microorganisms13010049

**Published:** 2024-12-30

**Authors:** Milena Samojlović, João R. Mesquita, Sérgio Santos-Silva, Malin Neptin, Joakim Esbjörnsson

**Affiliations:** 1 Systems Virology, Faculty of Medicine, Lund University, 223 62 Lund, Sweden; malin.neptin@med.lu.se (M.N.); joakim.esbjornsson@med.lu.se (J.E.); 2 Instituto de Ciências Biomédicas Abel Salazar, University of Porto, 4050-313 Porto, Portugal; jrmesquita@icbas.up.pt (J.R.M.); sergiosilva.1999@hotmail.com (S.S.-S.)

**Keywords:** one health approach, SARS-CoV-2, sheep, sentinels, pandemic preparedness

## Abstract

SARS-CoV-2 can cause clinical and inapparent disease and mortality in several animals cohabitating with humans, and sheep are susceptible to SARS-CoV-2 due to virus–receptor interactions similar to those in humans. Hence, sheep have the potential to be infected, spread, and develop neutralising antibodies (NAbs) against SARS-CoV-2. The aim of this study was to investigate the prevalence of SARS-CoV-2 NAbs in farm animals after natural exposure to the virus. Serum samples were collected from sheep in the Serra da Estrela region in Portugal, both prior to and during the COVID-19 pandemic. The sera were tested by established SARS-CoV-2 pseudovirus systems for multiple SARS-CoV-2 variants (early—Wuhan, mid—Delta, Omicron—BA.1, and late—Omicron XBB, BQ.1.1). Partial neutralisation activity in Pre-pandemic and Mid-pandemic samples was observed, while no NAb activity was observed in Late-pandemic samples tested. Different levels of NAbs were observed between Pre-pandemic samples and those collected during the Mid-pandemic and Late-pandemic periods (*p* ≤ 0.01). Our results indicate that SARS-CoV-2 cross-species transmission may have occurred through human–sheep contacts on sheep farms during the pandemic, and that farm animals could contribute to the One Health Approach in zoonotic virus surveillance and pandemic preparedness.

## 1. Introduction

Coronaviruses (CoVs) are a family of viruses that can infect multiple animal species, including birds, mammals, and humans, and with the potential to diversify and cross species barriers [1]. The recent SARS-CoV-2 pandemic (COVID-19) was caused by a coronavirus of zoonotic origin [2] and has led to vast global health problems and challenges, economic hardships, and social insecurities over the recent years. The emergence of new SARS-CoV-2 variants due to adaptation to the human host has resulted in more effective virus transmission and immune escape [3]. SARS-CoV-2 infection can lead to both severe disease and mortality in several animals cohabitating with humans [4,5]. So far, SARS-CoV-2 has been reported in 35 animal species from 16 different families (Felidae, Viverridae, Hyaenidae, Canidae, Mustelidae, Procyonidae, Cervidae, Hippopotamidae, Hominidae, Castoridae, Hyaenidae, Equidae, Cercopithecidae, Cebidae, Myrmecophagidae, and Cricetidae) [6,7]. Among the different circulating SARS-CoV-2 variants in animals, the Delta variant has been the most common and has been detected in 15 different animal species. Moreover, the Wuhan variant has been detected in eight different animal species, whereas ten different animal species have shown susceptibility to more than one SARS-CoV-2 variant [6].

Sentinel animals for SARS-CoV-2 could be any animal in which changes in different characteristics (e.g., levels of antibodies) can be measured to assess interrelationships between humans and animals. Hence, sentinel animals may serve as an early warning system for the emergence of new or altered viruses with zoonotic potential [8]. For example, wild ruminants, such as white-tailed deer, have been reported to be very susceptible to SARS-CoV-2 infection, being infected with four different variants: Alpha, Delta, Gamma, and Omicron [6,9,10]. Since deer-to-deer SARS-CoV-2 transmission can occur between white-tailed deer, an increased risk of deer-to-human spill-over has been suggested [11]. Although the susceptibility of sheep to SARS-CoV-2 infection differs from that in white-tailed deer, recent studies have shown that sheep are susceptible to SARS-CoV-2, both by virus replication in ovine-derived cell cultures and by experimental infection [12,13]. In addition, seroconversion and the production of neutralising antibodies (NAbs) towards SARS-CoV-2 have been reported in infected sheep by ELISA and/or a virus neutralisation test (VNT) [14,15]. However, the experimental exposure of animals to viruses may not always be comparable to the exposure in natural conditions, for example, in relation to entry routes, and the virus dose is typically higher in experimental conditions [16]. Following experimental SARS-CoV-2 infection, a higher number of seropositive sheep was detected by ELISA, while only one sheep had low levels of NAbs at a 1:20 titre value, detected by VNT [13]. Similar results were obtained after natural exposure to the virus, which was the first serological evidence of SARS-CoV-2 infection in sheep and goats [14]. On the other hand, Villaneuva-Saz et al. did not find any SARS-CoV-2 antibody positive samples in sheep that were in close contact with the veterinary student community in Spain during the pandemic [16].

Sheep farming in Portugal has a long tradition, especially in rural areas, which has resulted in local native sheep breeds suitable for either meat, milk, or wool [17]. One of the most important dairy sheep breeds is Serra da Estrela, inhabiting the Mountain region of the same name. The sheep are held in herds of different sizes, with an average herd size consisting of approximately 100 animals [18]. With a semi-intensive grazing regime, and milking performed two times per day, there are significant contacts between people and sheep as well as between grazing animals and potential wildlife on the farms in this region. Hence, SARS-CoV-2 virus cross-species transmission may have occurred through contacts between people and sheep during daily animal handling and milking procedures, as well as between gazing animals and susceptible wildlife during the SARS-CoV-2 pandemic. The aim of this study was to investigate the prevalence of SARS-CoV-2 NAbs in farm animals by analysing sheep serum collected in the Serra da Estrela region in Portugal both prior to and during the COVID-19 pandemic. More specifically, we have studied whether different SARS-CoV-2 variants circulating in people at certain time points had different influences on eliciting the NAb responses in sheep at corresponding time points.

## 2. Materials and Methods

### 2.1. Sample Collection and Study Region

Sheep serum samples were collected from the autochthonous sheep breed (Serra da Estrela) from dairy farms in Serra da Estrela Region, Central Portugal. The sheep included in the present study were kept in small herds (approximately 80 sheep per herd) and were in constant contact with farm workers, although no data were collected on whether some of the workers had COVID-19 infection during the pandemic or not. Official veterinarians for the national brucellosis surveillance program collected blood samples according to official regulations and protocols ensuring animal welfare [19]. Serum was collected from blood upon centrifugation (2500× *g*, 5 min), and then aliquoted and stored at −20 °C. In total, 180 serum samples were collected at three different periods: Pre-pandemic (N = 45) collected in 2016, Mid-pandemic (N = 58) collected in January 2022, and Late-pandemic (N = 77) collected in March 2023.

### 2.2. Pseudovirus Production and Pseudovirus Neutralisation Assay

HEK293T and HEK293T-ACE2 cells were cultured in Dulbecco’s Modified Eagle Medium (DMEM) (Gibco, Thermo Fisher Scientific, Inc., Waltham, MA, USA) with high glucose and sodium pyruvate, 10% foetal calf serum, and 100 IU/mL penicillin and 100 μg/mL streptomycin (Gibco, Thermo Fisher Scientific, Inc., MA, USA)—complete cell medium. The cells were propagated at 37 °C (5% CO_2_).

The presence and efficiency of NAbs towards SARS-CoV-2 in sheep serum were determined by a SARS-CoV-2 pseudovirus system for multiple SARS-CoV-2 variants. Briefly, pseudovirus constructs (PVs) were generated by three-plasmid co-transfection of HEK293T cells using a lentiviral backbone (Addgene, #8455), firefly luciferase reporter (Addgene, #170674), and S protein expressing plasmids of the SARS-CoV-2 variants (Wuhan, Delta, Omicron BA.1, XBB, and BQ.1.1 [Addgene, #170442, #172320, #180375, #194494, #194493]) (Table S1), using Lipofectamine 3000 (Invitrogen, Thermo Fisher Scientific, Inc., Waltham, MA, USA) as a transfection reagent [20,21,22,23,24]. A day prior to transfection, HEK293T cells were seeded in 6-well plates in a concentration of 5 × 10^5^/mL, aiming to achieve 70–80% confluency on the day of transfection. The total DNA to Lipofectamine 3000 ratio was 1:2 per well, following the manufacturer’s instructions. DNA–lipid complexes were incubated for 20 min at room temperature and added to the wells of 6-well plates with previously seeded HEK293 cells. Plates were incubated for 24 h at 37 °C (5% CO_2_), after which the cell medium was replaced with a new cell medium and incubated for an additional 36 h in the same conditions. The cell medium was collected 60 h after the transfection and then centrifuged for 7 min at 1500 rpm followed by filtering of the supernatant on a sterile 0.45 µm pore-sized filter (Thermo Fisher Scientific, Inc., Waltham, MA, USA). The filtrate of pseudovirus particles was then aliquoted in 1.5 mL cryotubes and frozen at −80 °C until use [25]. A vesicular stomatitis virus (VSV) pseudovirus was constructed by replacing the S protein expressing plasmid with the G envelope protein expressing plasmid pCMV-VSV-G (Cell Biolabs, Inc., San Diego, CA, USA) following the above-mentioned protocol. The VSV pseudoviruses were then used as positive controls for three-plasmid transfection efficacy. Prior to the neutralisation assay, an infectivity check and titration of collected pseudoviruses was conducted in HEK293T-ACE2 cells, followed by incubation of 72 h at 37 °C and luminescence measurement using the Bright-Glo Luciferase Assay System (Promega, Madison, WI, USA) in black 96-well plates (Corning Inc., Corning, NY, USA) using a VICTOR^®^ Nivo™ Plate Reader (PerkinElmer, Waltham, MA, USA).

The pseudovirus neutralisation assay was performed in flat bottom 96-well plates (TPP Techno Plastic Products AG, Trasadingen, Switzerland) in HEK293T-ACE2 cells, as described [25]. Briefly, all serum samples were inactivated at 56 °C for one hour prior to testing. Three-fold serially diluted sheep sera were tested in duplicates and incubated for 1 h at 37 °C with SARS-CoV-2 pseudoviruses capable of producing approximately 100,000 relative light units in 50 µL. Negative (cell medium) and positive (WHO standards) controls were included in all neutralisation experiments. After initial incubation and addition of 20,000 HEK293T-ACE2 cells per well, plates were incubated for 72 h at 37 °C. Luminescence was measured using the Bright-Glo Luciferase Assay System (Promega, Madison, WI, USA) in black 96-well plates (Corning Inc., Corning, NY, USA) using a VICTOR^®^ Nivo™ Plate Reader (PerkinElmer, Waltham, MA, USA).

### 2.3. Statistical Analysis

Neutralisation was quantified as the reduction in luciferase activity relative to the average of six control wells infected with pseudoviruses in the absence of serum and plotted against the logarithm of the dilution factors using GraphPad Prism 10.1.0 software (San Diego, CA, USA), as previously described [25]. A four-parameter logistic equation was then fitted to the neutralisation curves. The limit of detection (LOD) was calculated using the mean titre of the negative control samples +1.96 standard deviations, as described previously [25]. The non-parametric Kruskal–Wallis test, followed by post-hoc Dunn’s test, was used to test the null hypothesis “There is no difference in the level of antibody neutralisation between Wuhan and other tested SARS-CoV-2 variants in the Pre-pandemic, Mid-pandemic and Late-pandemic samples”. The results were considered statistically significant at *p* ≤ 0.01. Bonferroni correction was used to correct for multiple comparisons.

## 3. Results

**Figure 1:**
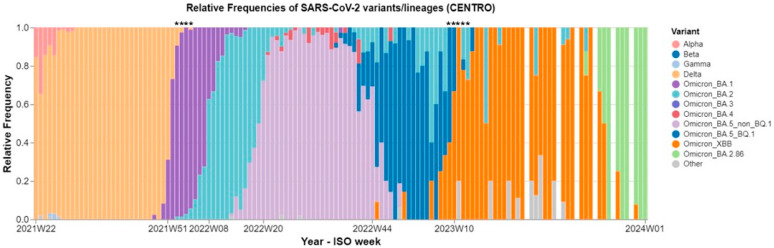
Overview of weekly relative frequency of SARS-CoV-2 variants during 2021–2023 in Central Portugal. In the period of first pandemic sampling in January 2022 (week 1 to week 4—black stars ****), the most dominant SARS-CoV-2 variant circulating was Omicron BA.1, preceded by the Delta variant. In the period of second pandemic sampling in March 2023 (week 9 to week 13—black stars *****), the most dominant variant circulating was Omicron XBB, preceded by Omicron BA.5_BQ.1 [26].

Pre-pandemic samples (N = 45) collected in 2016 were tested for the presence of NAbs towards the SARS-CoV-2 Wuhan variant. Mid-pandemic samples collected during January 2022 were tested towards the SARS-CoV-2 Delta (N = 30) and Omicron BA.1 (N = 28) variants, whereas Late-pandemic samples collected in March 2023 were tested towards the XBB (N = 47) and BQ.1.1 (N = 30) variants, respectively. Hence, pandemic samples were tested against variants circulating in the human population in Portugal just before or during the period of sampling (Figure 1) [26]. The most common circulating SARS-CoV-2 variant in January 2022 (week 1 to week 4) was Omicron BA.1 (preceded by the Delta variant), while the most common variant in March 2023 (week 9 to week 13) was Omicron XBB (preceded by the Omicron BA.5_BQ1 variant). The cut-off value of neutralisation was calculated to 61%, taking the higher non-specific background of animal sera into consideration [27].

**Figure 2:**
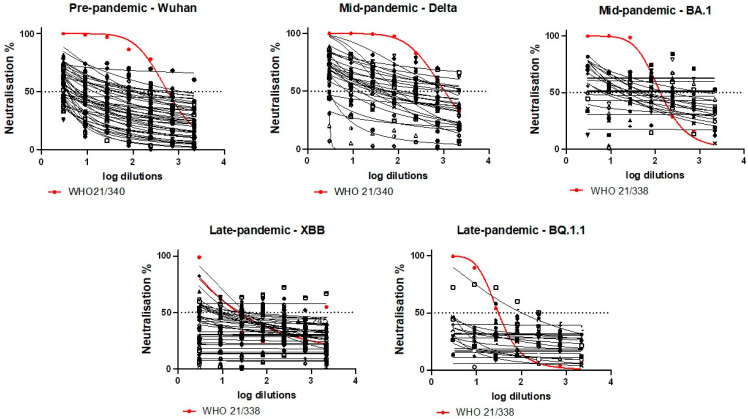
Neutralisation curves of SARS-CoV-2 pseudovirus variants by sheep sera in HEK293T-ACE2 cells. Sera were collected Pre-pandemic (N = 45) and during pandemic (Mid-pandemic January 2022, N = 58 and Late-pandemic March 2023, N = 77) and tested by pseudovirus neutralisation assays towards earliest SARS-CoV-2 or SARS-CoV-2 variants circulating at sampling time (black lines and dots), as well as WHO International Standards for anti-SARS-CoV-2 immunoglobulin for early (21/340) and Omicron (21/338) variants (red lines and dots).

The differences in the neutralisation response curves between Pre-pandemic, Mid-pandemic, and Late-pandemic samples, as well as between different SARS-CoV-2 variants, were assessed with sheep sera and WHO anti-SARS-CoV-2 antibody standards (Figure 2). At high concentrations, the WHO standard for anti-SARS-CoV-2 immunoglobulin for early variants (21/340) neutralised the Wuhan and Delta PVs, and the anti-SARS-CoV-2 immunoglobulin for Omicron variant (21/338) neutralised the Omicron BA.1 and BQ.1.1 PVs. However, the Omicron XBB PVs were not well neutralised by the WHO standard for anti-SARS-CoV-2 immunoglobulin for Omicron variant (21/338).

Although most sheep sera showed lower neutralisation efficiency compared with the WHO controls, 10% of the Mid-pandemic samples had close to 100% neutralisation efficiency towards the SARS-CoV-2 Delta variant when undiluted (Figure 2). Moreover, 40% of the Pre-pandemic sera had a neutralisation efficiency above the limit of detection (LOD), with one sample having 81% neutralisation efficiency towards the Wuhan PVs in the lowest dilution (1:3). The other Pre-pandemic sera had a neutralisation efficiency between 60–80%, with neutralisation percentage gradually decreasing with increased dilution. Among all samples tested, Mid-pandemic samples had the highest proportion (23%) of over 80% neutralisation efficiency against Delta PVs at the lowest dilution level, compared to the Pre-pandemic and Late-pandemic samples tested against Wuhan and Omicron PVs, respectively. Moreover, 70% of Mid-pandemic sera tested against the Delta variant had neutralisation levels above the LOD. In contrast, only 32% of Mid-pandemic sera showed detectable neutralisation activity above the LOD against Omicron BA.1. The neutralisation efficiency dropped significantly for Late-pandemic sera, with only 6% and 3% of samples showing neutralisation activity above the LOD against Omicron XBB and BQ.1.1, respectively (Figure 2).

**Figure 3:**
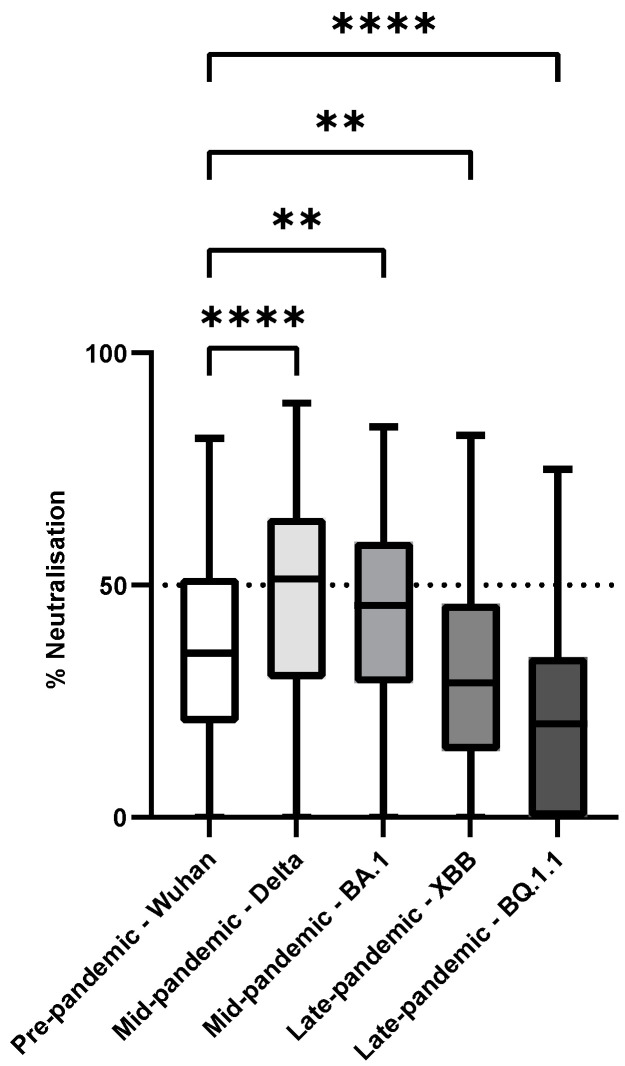
Comparison of sheep neutralising antibody levels across different SARS-CoV-2 variants and across different sampling time periods by non-parametric Kruskal–Wallis multiple comparison test, followed by post-hoc Dunn’s test (** = *p* ≤ 0.01, **** = *p* ≤ 0.0001).

The median neutralisation percentage (IQR) of the tested SARS-CoV-2 variants was 36% (20–52%) for the Wuhan variant, 51% (30–64%) for the Delta variant, 46% (29–59%) for the BA.1 variant, 29% (14–46%) for the XBB variant, and 20% (0–34%) for the BQ.1.1 variant (Figure 3). Interestingly, the neutralisation effectiveness was significantly higher among the Mid-pandemic Delta and BA.1 variants compared with the Wuhan variant (*p* < 0.0001 and *p* = 0.0013, respectively). In contrast, the neutralisation effectiveness was lower among the Late-pandemic XBB and BQ.1.1 variants compared with the Wuhan variant (*p* = 0.0034 and *p* < 0.0001, respectively).

## 4. Discussion

In the present study, we investigated the seroprevalence of NAbs towards different SARS-CoV-2 variants in sheep sera collected before and during the SARS-CoV-2 pandemic and observed different longitudinal trends in NAb prevalence depending on sampling time and tested SARS-CoV-2 variants. Since antibody screening has been suggested as an important tool for the detection of infected animals, due to a longer detection window of circulating antibodies compared to direct virus identification, we assessed if sheep could serve as an indirect indicator for SARS-CoV-2 virus circulation in humans [28].

Overall, the tested sheep serum samples only partially neutralised the different assessed SARS-CoV-2 variants, irrespective of whether they were collected before and after possible natural exposure. However, the neutralisation effectiveness across variants differed, indicating higher neutralisation efficiencies of sera collected Mid-pandemic (Delta and BA.1 variants) compared with Late-pandemic (XBB and BQ1.1 Omicron variants). In addition, the neutralisation efficiencies of sera collected Mid-pandemic were more similar to the Pre-pandemic sera (Wuhan variant) compared with Late-pandemic variants. In line with this, antibody responses towards the Delta variant in humans have been suggested to be more similar to responses elicited by early 2020 variants, such as the Wuhan variant [29]. In addition, antibody escape was recently shown for SARS-CoV-2 Omicron variants due to the newly introduced mutations in spike protein [30,31]. Moreover, we observed an increase in the neutralising antibody efficiency between samples collected Pre-pandemic and Mid-pandemic. Similar trends were reported in a study in cattle [32], indicating that Portuguese sheep from our study may have been exposed to SARS-CoV-2 during the pandemic, in particular to the early SARS-CoV-2 Delta variant, but to a lesser extent to the Omicron variants BA.1, XBB, and BQ.1.1.

Utilizing a variant-specific SARS-CoV-2 pseudovirus neutralisation assay lowers the possibility of cross-reactivity to other SARS-CoV-2 variants or non-targeted coronaviruses due to specific epitope mutations, a phenomenon that has been particularly observed in Omicron variants and that may influence antibody response [25,33]. However, a factor that could impact the accuracy of the determined NAb efficiency in SARS-CoV-2 pseudovirus-based neutralisation assays is the non-specific background of human and animal negative sera seen at lower dilutions [27]. A higher background was also observed in Pre-pandemic healthy donor sera tested against SARS-CoV-2 in a cell- and virus-free S protein-based neutralisation assay [34]. Hence, we cannot exclude a high background of sheep sera due to the potential previous exposure of sheep-to-bovine coronavirus in our study. Indeed, some cross-reactivity between SARS-CoV-2 Nabs and bovine coronavirus has been reported, particularly towards early SARS-CoV-2 variants [35].

In summary, we observed an increase in neutralising antibody levels during the pandemic, in particular towards early SARS-CoV-2 variants, where spike mutations have been suggested to interfere less with antibody neutralisation [36]. Our results suggest that sheep may play a role as indirect indicators of SARS-CoV-2 infection in the human population and highlighting the importance of field studies and serological monitoring of virus circulation in natural conditions in different animal species. However, and although it has been suggested that potential SARS-CoV-2 virus cross-species transmission could have been established through contacts between people and sheep during the pandemic, it is important to emphasize that further studies are needed to determine the impact of potential sheep-to-sheep and sheep-to-human transmissions. Finally, whilst this study does not directly address One Health challenges like disease outbreak and surveillance on a large scale, the results highlight that interdisciplinary collaboration and enhanced disease monitoring are important in addressing challenges posed by zoonotic viruses [37,38,39]. An essential part of pandemic preparedness is to create more rapid responses between pathogen emergence and development of diagnostics and medical countermeasures, as well as implementing research outcomes effectively in both the human and the animal population [40,41].

## 5. Conclusions

Using animals cohabiting in close contact with humans as sentinels has both potential benefits and limitations for zoonotic virus surveillance, offering insights into high-risk transmission areas while also presenting challenges in reliability and effectiveness. In our study, we identified partial neutralisation activity of Pre-pandemic sheep sera against the earliest SARS-CoV-2 variant Wuhan. Mid- and Late-pandemic sheep sera were assessed against the variants circulating at the time of sampling. Mid-pandemic sera showed partial neutralisation activity towards Delta and BA.1 variants, whereas Late-pandemic sera showed no neutralisation activity towards the SARS-CoV-2 Omicron variants XBB and BQ.1.1. Our results suggest that SARS-CoV-2 species spill-over events from humans to sheep may have occurred on sheep farms during the pandemic, indicating that farm animals may play an important role as sentinels for zoonotic virus surveillance. Taken together, our study supports the broader concept of the One Health Approach as an important tool for future pandemic preparedness.

## Data Availability

The authors confirm that the data supporting the findings of this study are available within the article.

## Supplementary Materials

The following supporting information can be downloaded at: https://www.mdpi.com/article/10.3390/microorganisms13010049/s1, Table S1: Information on Spike expressing plasmids of different SARS-CoV-2 variants used for the production of SARS-CoV-2 pseudoviruses.
