# Supplementary material for: Investigating SARS-CoV-2 Neutralising Antibody Response in Sheep
Source: Microorganisms. 2024 Dec 30;13(1):49. doi: 10.3390/microorganisms13010049 (PMC11767788; doi:10.3390/microorganisms13010049)
Supplement: Supplementary file 1 [file microorganisms-13-00049-s001.zip › microorganisms-3358990-supplementary.pdf]

Table S1. Information on Spike expressing plasmids of different SARS-CoV-2 variants used for the production of SARS-CoV-2 pseudoviruses. More information on the following plasmids can be found on www.addgene.com, or in the referenced publications.

No.	Plasmid name	Addgene number	SARS-CoV-2 Variant	GenBank ID	Species/strain	Mutations	Deposition Lab	Reference
1	pcDNA3.3_CoV2_D18	170442	Wuhan	43740568	SARS-CoV-2, USA-WA1/2020	18 aa1 deletion in c-terminal tail	David Nemazee	[21]
2	pcDNA3.3-SARS2-B.1.617.2	172320	Delta	43740568	SARS-CoV-2, B.1.617.2	T19R, 156G, 157-158del, L452R, T478K, D614G, P681R, D950N, c-terminal 18 aa deletion	David Nemazee	[22]
3	pcDNA3.3_SARS2_omicron_BA.1	180375	Omicron BA.1	UFO69279	SARS-CoV-2, Omicron BA.1	C terminal 18 aa deletion	David Nemazee	[23]
4	pcDNA3.3_SARS2_XBB	194494	Omicron XBB	N/A	SARS-CoV-2, Omicron XBB	V83A, Y144-, H146Q, Q183E, V213E, G339H, L368I, R346T, V445P, G446S, N460K, F486S, F490S, R493Q, and c-terminal 18 aa deletion on the basis of BA.2	David Nemazee	[24]
5	pcDNA3.3_SARS2_BQ.1.1	194493	Omicron BQ.1.1	N/A	SARS-CoV-2, Omicron BQ.1.1	R346T, K444T, N460K and c-terminal 18 aa deletion on the basis of BA.5	David Nemazee	[24]

1 aa – amino acids

References:

21. Rogers, T.F.; Zhao, F.; Huang, D.; Beutler, N.; Burns, A.; He, W.T.; Limbo, O.; Smith, C.; Song, G.; Woehl, J.; Yang, L.; Abbott, R.K.; Callaghan, S.; Garcia, E.; Hurtado, J.; Parren, M.; Peng, L.; Ramirez, S.; Ricketts, J.; Ricciardi, M.J.; Rawlings, S.A.; Wu, N.C.; Yuan, M.; Smith, D.M.; Nemazee, D.; Teijaro, J.R.; Voss, J.E.; Wilson, I.A.; Andrabi, R.; Briney, B.; Landais, E.; Sok, D.; Jardine, J.G.; Burton, D.R. Isolation of potent SARS-CoV-2 neutralizing antibodies and protection from disease in a small animal model. Science 2020, 69(6506), 956-963.
22. Cho, H.; Gonzales-Wartz, K.K.; Huang, D.; Yuan, M.; Peterson, M.; Liang, J.; Beutler, N.; Torres, J.L.; Cong, Y.; Postnikova, E.; Bangaru, S.; Talana, C.A.; Shi, W.; Yang, E.S.; Zhang, Y.; Leung, K.; Wang, L.; Peng, L.; Skinner, J.; Li, S.; Wu, N.C.; Liu, H.; Dacon, C.; Moyer, T.; Cohen, M.; Zhao, M.; Lee, FE.; Weinberg, R.S.; Douagi, I.; Gross, R.; Schmaljohn, C.; Pegu, A.; Mascola, J.R.; Holbrook, M.; Nemazee, D.; Rogers, T.F.; Ward, A.B.; Wilson, I.A.; Crompton, P.D.; Tan, J. Bispecific antibodies targeting distinct regions of the spike protein potently neutralize SARS-CoV-2 variants of concern. Sci Transl Med 2021, 13(616), eabj5413.
23. Dacon, C.; Tucker, C.; Peng, L.; Lee, C.D.; Lin, T.H.; Yuan, M.; Cong, Y.; Wang, L.; Purser, L.; Williams, J.K.; Pyo, C.W.; Kosik, I.; Hu, Z.; Zhao, M.; Mohan, D.; Cooper, A.J.R.; Peterson, M.; Skinner, J.; Dixit, S.; Kollins, E.; Huzella, L.; Perry, D.; Byrum, R.; Lembirik, S.; Drawbaugh, D.; Eaton, B.; Zhang, Y.; Yang, E.S.; Chen, M.; Leung, K.; Weinberg, R.S.; Pegu, A.; Geraghty, D.E.; Davidson, E.; Douagi, I.; Moir, S.; Yewdell, J.W.; Schmaljohn, C.; Crompton, P.D.; Holbrook, M.R.; Nemazee, D.; Mascola, J.R.; Wilson, I.A.; Tan, J. Broadly neutralizing antibodies target the coronavirus fusion peptide. Science 2022; 377(6607), 728-735.
24. Zhou, P.; Song, G.; Liu, H.; Yuan, M.; He, W.T.; Beutler, N.; Zhu, X.; Tse, L.V.; Martinez, D.R.; Schäfer, A.; Anzanello, F.; Yong, P.; Peng, L.; Dueker, K.; Musharrafieh, R.; Callaghan, S.; Capozzola, T.; Limbo, O.; Parren, M.; Garcia, E.; Rawlings, S.A.; Smith, D.M.; Nemazee, D.; Jardine, J.G.; Safonova, Y.; Briney, B.; Rogers, T.F.; Wilson, I.A.; Baric, R.S.; Gralinski, L.E.; Burton, D.R.; Andrabi, R. Broadly neutralizing anti-S2 antibodies protect against all three human betacoronaviruses that cause deadly disease. *Immunity* **2023**, *56*(3), 669-686.e7.
